# TriLex: A fusion approach for unsupervised sentiment analysis of short texts

**DOI:** 10.1371/journal.pone.0317100

**Published:** 2025-04-17

**Authors:** Abdulrahman Alharbi, Rafaa Aljurbua, Shelly Gupta, Zoran Obradovic

**Affiliations:** 1 Department of Computer and Information Sciences, Temple University, Philadelphia, Pennsylvania, United States of America; 2 Department of Computer Science, College of Engineering and Computer Science, Jazan University, Jazan, Saudi Arabia; 3 Department of Computer Science, College of Computer, Qassim University, Buraydah, Saudi Arabia; Mehran University of Engineering & Technology, PAKISTAN

## Abstract

In recent years, online customer reviews and social media platforms have significantly impacted individuals’ daily lives. Despite the generally short nature of textual content on these platforms, they convey a wide range of user sentiments. However, sentiment analysis of short texts poses a challenge due to their context limitations. In addition, traditional supervised machine learning methods often struggle with the dynamic nature of sentiment expression and the scarcity of labeled data, which is a cost-efficiency issue. To address these challenges, this paper proposes TriLex, a novel unsupervised approach that leverages the majority votes of multiple lexicon-based sentiment analysis tools. TriLex categorizes agreement among TextBlob, VADER, and AFINN as strong labels and disagreement as weak labels. To improve sentiment labeling, we normalize sentiment scores across all lexicons and apply weighted averaging to compute a majority vote sentiment score. It then generates a new label for the weak label based on a dynamic threshold derived from the majority vote. The effectiveness of TriLex is evaluated on benchmark datasets for the accuracy, F1 score, precision, and recall of Logistic Regression, XGBoost, and LSTM models. The proposed TriLex model improves the accuracy of sentiment prediction by 2%–8%. Overall, our results demonstrate that TriLex outperformed methods relying on individual lexicons and existing fusion-based alternatives.

## 1 Introduction

Sentiment analysis is a branch of artificial intelligence that uses Natural Language Processing (NLP) and machine learning techniques to study people’s emotions and opinions through computational methods [1,2]. It has emerged as a powerful tool that automates the mining of attitudes, opinions, views, and emotions from text, speech, and tweets [3]. This capability is critical for understanding public sentiment in various domains, such as social media [4], product review [5], and healthcare [6]. Sentiment analysis involves classifying opinions in the text as positive, negative, and neutral. It is also referred to as subjectivity analysis, opinion mining, and appraisal extraction. To achieve accurate sentiment classification, different advanced approaches have been developed.

There are various approaches to interpreting and classifying emotions in text, including lexicon-based [7–9], machine learning [10–12], and deep learning [13–17]. Traditional methods include lexicon-based techniques, where words are assigned sentiment scores based on predefined dictionaries. However, more sophisticated methods have emerged with the advent of machine learning. Supervised machine learning approaches often require labeled datasets to train models that can classify sentiment. Further, deep learning techniques, such as neural networks, can capture complex patterns and nuances in language. Despite these advancements in sentiment analysis, short texts remain a significant challenge.

In the field of sentiment analysis, researchers have explored supervised, semi-supervised, and unsupervised techniques to tackle the challenge of accurately determining sentiment in text.

Supervised sentiment analysis techniques have shown promising results in recent years. However, these approaches require high-quality manual labeled data for training, which is often scarce and expensive to obtain [18,19] when it comes to new domains, especially for short texts. Moreover, supervised models often struggle to generalize across different domains, resulting in poor performance when applied to another domain. Another challenge is the requirement of long training time, huge datasets, and storage availability for machine learning and deep learning models [20–22].

Semi-supervised techniques aim to leverage both labeled and unlabeled data to improve sentiment analysis performance. Chamid et al. [23] proposed a graph-based semi-supervised approach for Indonesian aspect-based sentiment analysis, using Graph Convolutional Networks (GCN) and Graph Recurrent Networks (GRN) to detect aspect and opinion relationships. Although this method shows promise in reducing the need for extensive labeled data, it still faces challenges in handling noisy labels and maintaining consistent performance across different domains. Li et al. [24] introduced a dual consistency-enhanced semi-supervised network for sentiment analysis of COVID-19 tweets, addressing the limited annotated training dataset issue. However, semi-supervised methods often rely on assumptions about the distribution of unlabeled data, which may not hold true in all cases, potentially leading to degraded performance in certain scenarios.

Unsupervised sentiment analysis methods, which do not rely on labeled data, have emerged as a promising solution for short-text analysis due to their ability to operate without labeled data. These methods typically leverage lexicon-based techniques, assigning sentiment scores to individual words or phrases based on pre-defined sentiment lexicons [25]. However, existing lexicon-based approaches often suffer from limited coverage, domain specificity, and the inability to handle context-dependent sentiment expressions.

Sentiment analysis of short text, such as social media posts, product reviews, and customer feedback is challenging due to the limited context and informal language used. In our paper, we address one of the gaps and limitations in the field of sentiment analysis discussed in a recent survey [26]. The limitation of supervised methods is the cost efficiency of the dependency on humans manually labeling a large dataset. More recently, unsupervised methods have gained traction, particularly for short texts with scarce labeled data. These methods leverage advancements in natural language processing to infer sentiment without direct human annotation, offering a promising direction for sentiment analysis. Therefore, we follow the unsupervised approach to address the limitations related to sentiment analysis for short text.

Despite the extensive use of lexicon-based approaches, sentiment analysis often relies on a single lexicon, which can limit its effectiveness due to the static nature of the lexicon and the lack of contextual adaptability. To address these challenges and limitations, we propose TriLex, a novel unsupervised approach that combines the strengths of three widely used lexicons: TextBlob [27], Valence Aware Dictionary for sEntiment Reasoner (VADER) [28], and AFINN [29]. This integration is grounded in the concept of ensemble methods. TriLex takes advantage of the diverse linguistic features captured by these lexicons and combines their outputs to enhance the accuracy and reliability of sentiment analysis for short texts. By drawing on these theoretical foundations, we aim to bridge gaps in existing sentiment analysis techniques and contribute to advancing the field’s understanding of how lexicon integration can enhance both the granularity and adaptability of sentiment scoring for short text. Expanding on these theoretical frameworks, we aim to clarify the significance of our work and its potential to address key challenges in sentiment analysis for a large corpus of short text when labels are limited.

Each lexicon has unique attributes that contribute differently to sentiment scoring. Therefore, we introduce a dynamic threshold mechanism that adjusts to the sentiment distribution within each domain, allowing our system to classify sentiments with higher precision. In cases where lexicons disagree, we normalize the sentiment score and average the lexicon weight to compute composite sentiment scores and generate an enhanced single sentiment label. The proposed approach outlines how multi-lexicon integration, dynamic thresholding, and composite scoring mechanisms contribute to a more sophisticated understanding of sentiment classification for short text. It represents a novel approach that improves the adaptability and accuracy of lexicon-based sentiment analysis.

The objective of our paper is to determine whether the proposed TriLex methodology could improve the labeling of public sentiment in a short text. Therefore, we evaluated its competitive performance on publicly available datasets, including the COVID-19 Vaccines dataset from Kaggle [30,31], Sentiment140 [32], Amazon Review [33], and US Airline Tweets from Kaggle. The main contributions of our paper are summarized as follows:

1. Develop an enhanced TriLex lexicon approach integrating three lexicons: TextBlob, VADER, and AFINN based on a majority vote. The majority vote agreement is considered as a strong label, and disagreement as a weak label. We assign new labels to weak labels by assigning weights to each lexicon to compute the composite sentiment score and then categorize the sentiment based on a new threshold. This method aimed to determine the most effective technique for offering valuable insights into sentiment trends.
2. Enhance sentiment labeling by encompassing normalized sentiment scores across lexicons, applying weighted averaging, and introducing an effective polarity categorization mechanism to calculate the composite sentiment score.
3. Leverage the collective features of multiple lexicons to effectively capture a wider range of sentiment patterns and expressions compared to individual lexicons.

The remainder of this paper is organized as follows. Sect 2 discusses related previous studies. Sect 3 describes the methodology, including datasets, preprocessing, lexicons integration and normalization, and sentiment categorization approach. Sect 4 shows the obtained results, findings, and discussions. Finally, Sect 5 presents the conclusions and future works.

## 2 Related works

Sentiment analysis is a rapidly growing sub-field in natural language processing. Social media platforms have become increasingly popular tools for communication. As a result of this growth, social media platforms have become crucial sources of information, thus contributing to increased availability of data for analysis [34].

Researchers often formulate the sentiment analysis problem using machine learning models such as Support Vector Machine, Decision Tree, Logistic Regression, and Naive Bayes [35–37]. Prior studies have adopted deep learning algorithms with the goal of designing black box models that provide enhanced sentiment prediction accuracy. The Dynamic Convolutional Neural Network (DCNN) is introduced for sentiment classification of movie reviews and Twitter data [38]. The DCNN employs dynamic k-Max pooling to effectively handle sentences with varying lengths. A GloVe-DCNN model is introduced that performs the binary classification of tweets into either negative or positive sentiment categories [39].

Lexicon-based methods are common approaches for sentiment analysis, which automatically detect a text’s emotional tone or attitude. These approaches rely on a predefined list of words or phrases associated with different sentiments and assign them a polarity score based on their sentiment. Researchers evaluate the sentiment of a text by leveraging pre-existing lexicons of words, each with assigned sentiment weights such as [40,41]. A sentiment analysis system is developed for Twitter called TwiSent [42]. TwiSent gathered tweets related to the searched topic and classified them into different polarity categories: positive, negative, and objective. SentiCircles is a promising approach that considers the contextual and conceptual semantics of words when determining their sentiment orientation and strength [43].

In a related context, TextBlob, VADER, and AFINN are commonly used lexicon-based approaches for sentiment analysis. However, they differ in their underlying approaches and methodologies. TextBlob and VADER are rule-based methods that rely on predefined lexicons and sets of rules to determine sentiment. TextBlob is a Python library offering a simple sentiment analysis API using a pattern-based approach. It assigns polarity scores (ranging from –1 to 1) to text based on the sentiment scores of words present and additional rules [27,44]. VADER is another widely used sentiment analysis tool specifically designed to handle social media text. It uses a rule-based approach to provide a sentiment intensity score that ranges from –1 to  + 1 [28,44]. In contrast, AFINN is a polarity-based method that is designed to analyze microblogs’ sentiment. It relies on a list of words rated for sentiment polarity. The words in the text are assigned scores ranging from –5 (negative) to  + 5 (positive), and calculates the sentiment score of a text by summing up the valence scores of individual words present. Thus, the polarity score is calculated based on the normalized score to a range of (–1 to  + 1) [29]. While VADER incorporates additional rules specific to social media language, making it more suitable for analyzing informal online text, TextBlob and AFINN are more general-purpose sentiment analysis tools. Comparing the performance of lexicon-based approaches on Twitter datasets have been addressed in the literature. For instance, in one study, the performance of different lexicon-based sentiments is compared and is concluded that AFINN obtained the highest accuracy [45], while in another study VADER achieved the best performance [46].

Recently, researchers have explored the effectiveness of unsupervised sentiment analysis to address the limitations of supervised approaches, particularly the dependency on labeled datasets. Punetha and Jain [19,47] introduced mathematical optimization models based on game theory to develop unsupervised models for sentiment classification in restaurant and product reviews. Their approaches enhance sentiment accuracy but rely on the availability of rating scores as part of the sentiment context, which limits their applicability in contexts where ratings are absent, such as in social media posts. Furthermore, these models classify sentiment into only positive and negative without accounting for neutral sentiment. This binary classification approach reduces their generalizability by neglecting neutral sentiments, which are prevalent in real-world data.

Other researchers have explored distinct methodologies to address sentiment ambiguity and domain adaptability. Vashishtha and Susan [48] combined the SentiWordNet lexicon with fuzzy linguistic techniques to analyze movie reviews, aiming to handle linguistic uncertainties. Despite promising results, this approach faces challenges with interpretability and generalizability beyond the movie domain, and the complexity of fuzzy rules introduces high computational demands on large datasets. Al-Ghuribi et al.[49] focused on Aspect-Based Sentiment Analysis (ABSA) by developing a hybrid model that integrates frequency-based, syntactic-relation, and semantic similarity methods for large-scale datasets. Their method assigns weights to extracted aspects using a modified TF-IDF scheme and derives aspect ratings from a domain-specific lexicon. Despite the valuable achievements of this approach, the lack of a neutral category reduces its ability to capture nuanced sentiment and domain-specificity. In a graph-based approach, Fares et al. [50] introduced the LISA framework, which leverages shortest-path techniques in a lexical-affective graph (LAG) to derive emotion categories such as anger, fear, and joy through unsupervised technique. However, the LISA approach requires substantial data and computational resources, posing scalability challenges. These studies highlight the diversity of unsupervised sentiment analysis techniques, each offering solutions tailored to specific challenges but also facing limitations regarding domain adaptability, interpretability, and computational efficiency.

Hybrid approaches have emerged as popular tools in sentiment analysis, enhancing performance in sentiment analysis tasks. For instance, a hybrid model is employed that consists of the hierarchical combination of Support Vector Machine and Random Forest [51]. In addition, a hybrid approach is introduced that integrates Gradient Boosting with SentiCircle for context-aware textual sentiment analysis [52]. Also, a hybrid method is proposed which combines text and image analysis [53]. Twitter provides a valuable source extensively employed in numerous sentiment analysis research studies. For instance, in one study, Twitter’s posts were classified as positive, negative, and neutral combing two models: NLP and a supervised K-Nearest Neighbors (KNN) algorithm [54] while another study combined VADER and TextBlob along with a pre-trained BERT [55]. In addition, TextBlob is applied with TF-IDF and LinearSVC to analyze public sentiments regarding COVID-19 vaccine hesitancy [56].

Recent studies have compared various word embedding techniques for sentiment analysis. The performance of TF-IDF, Word2vec, sent2vec, and BERT text embeddings are evaluated for sentiment analysis of online consumer reviews in retail sectors [57]. The evaluation showed that BERT and sent2vec produced stable results for predicting review ratings, while word2vec was more effective for identifying negative sentiment. Another study [58] provides an overview of text representation methods for sentiment analysis, including static approaches like TF-IDF and dynamic approaches like Word2Vec and BERT. The study found that contextualized embeddings like BERT consider word context, providing more nuanced representations than static approaches. Souza and Filho [59] explored the effectiveness of BERT and TF-IDF in sentiment analysis for Brazilian Portuguese and confirmed that BERT outperforms TF-IDF, but TF-IDF is a good trade-off between performance and cost. The effectiveness of embedding techniques varies across different applications and datasets in sentiment analysis.

**Fig 1:**
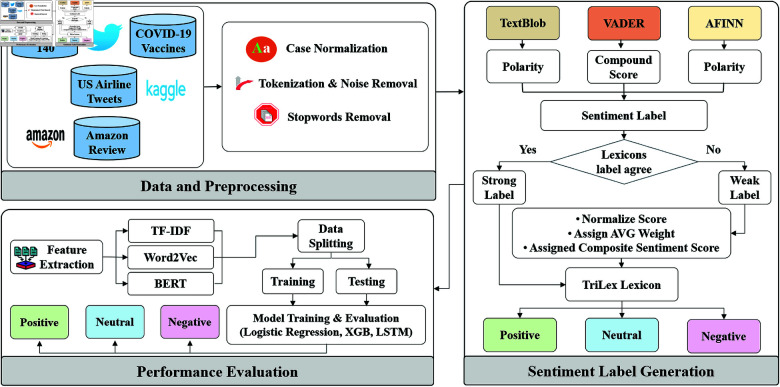
The workflow of the proposed TriLex unsupervised sentiment analysis approach to predict sentiment for short text.

Our study aims to accurately address sentiment analysis of short texts by adopting the unsupervised approach. Further, our proposed approach TriLex introduced to addresses several limitations seen in prior work including generalizability, cost-efficiency, and time-consuming. TriLex distinguishes itself by leveraging the collective features of multiple lexicons to effectively capture a broader range of sentiment patterns and expressions, and employing a dynamic threshold mechanism to adapt to the desired domain. Moreover, the proposed TriLex approach integrates TextBlob, VADER, and AFINN, using a majority vote and weighted averaging to compute composite sentiment scores and effectively categorize sentiments. It enhances weak labels by assigning weights to each lexicon to calculate a composite sentiment score. It then categorizes the sentiment based on a new threshold into positive, neutral, and negative. Also, it can be used to classify the sentiment into positive and negative only. To assess the TriLex performance against individual lexicons, four datasets were included in the final analysis: COVID-19 Vaccines, Sentiment140, Amazon Review, and the US Airline Tweets dataset. Our findings demonstrate that TriLex effectively categorizes sentiment with higher accuracy, improving generalizability and adaptability in a variety of contexts.

## 3 Materials and methods

The proposed system architecture for the unsupervised TriLex framework illustrated in Fig 1 consists of three main sections: Data and Preprocessing, Sentiment Label Generation, and Performance Evaluation. This architecture follows a structured workflow from raw input text to final sentiment classification output. It begins with data input and collection from various domains utilizing benchmark datasets, such as COVID-19 vaccine tweets, Sentiment140, Amazon reviews, and US airline tweets. These datasets undergo preprocessing and are transformed using word embedding techniques to extract relevant domain-specific features from the text. Particularly, we leverage TF-IDF vectorization, Word2Vec, and BERT embedding. In the Sentiment Label Generation phase, preliminary sentiment labels are assigned using baseline lexicons. The system then applies a majority voting technique to identify strong and weak labels, with a novel thresholding mechanism introduced for weak labels. This mechanism normalizes sentiment scores and averages weights across the baseline lexicons to enhance labeling accuracy.

To evaluate the performance of TriLex across individual lexicons, we split each dataset into training 80*%* and testing 20*%*. Then, we apply machine learning and deep learning models: Logistic Regression, XGBoost, and Long short-term memory (LSTM) to predict sentiment categories. The following subsections provide a detailed explanation of each step in the framework.

### 3.1 Datasets description

In this study, we evaluate the proposed methodology on the COVID-19 Vaccines dataset and three other widely used benchmark datasets, called Sentiment140, Amazon Review, and US Airline Tweets Sentiment.

#### 3.1.1 COVID-19 vaccines dataset.

The COVID-19 Vaccines dataset integrated two COVID-19 Vaccines tweets datasets obtained from Kaggle [30,31]. The integrated dataset contains 576 , 879 unique English tweets related to COVID-19 vaccines collected from August$1^{\text{st}}$, 2020 to February $19^{\text{th}}$, 2022. The dataset is unsupervised and has no sentiment label. Thus, we assign a sentiment label employing the proposed TriLex approach.

#### 3.1.2 Sentiment140 dataset.

The Sentiment140 dataset is a large collection of 1 . 6 million tweets, each labeled with a sentiment score ranging from 0 (negative) to 4 (positive). This dataset was created by researchers at Stanford University [32] and is widely used to evaluate sentiment analysis models on Twitter data. The tweets cover a diverse range of topics and represent a substantial challenge due to the volume of data and the inherent noise and ambiguity present in social media text. However, the publicly available dataset does not contain neutral sentiment, with 800*K* sentiments being positive and the other half being negative.

#### 3.1.3 Amazon review dataset.

The Amazon Review dataset is a corpus of 8 , 000 product reviews spanning multiple categories, such as books, DVDs, electronics, and kitchens [33]. Each review is paired with a rating ranging from 1 (negative) to 5 (positive) stars, which can be interpreted as a sentiment label. The publicly available dataset contains 1 , 000 positive and 1 , 000 negative reviews in each domain categorized based on rating score. This dataset provides a valuable resource for evaluating sentiment analysis techniques in the context of product reviews and customer feedback. Sometimes, the rating score does not reflect the sentiment; therefore, we regenerate the sentiment category based on the review text content by applying the TriLex approach.

#### 3.1.4 US airline tweets sentiment dataset

This dataset consists of 1 , 4640 tweets related to major US airlines, manually annotated with positive (16*%*), negative (63*%*), and neutral (21*%*) sentiment labels. The tweets were collected from February 2015 and covered a range of topics, including flight experiences, customer service, and airline policies. The dataset provides a test case for sentiment analysis on social media data, where informal language, abbreviations, and context-dependent expressions are prevalent.

### 3.2 Data preprocessing

The preprocessing of unstructured short text is a critical step in sentiment analysis to improve understanding of sentiments expression and emotion, and extracting meaningful insights from the original dataset. social media. This process is essential for eliminating noise and ambiguities; therefore, we perform data cleaning by removing punctuation, square brackets, punctuation numbers, and new lines and converting text to lowercase to ensure consistency. Also, we remove irrelevant content such as retweets, mentions, URLs, hashtags, symbols, numbers, and excess spaces. Additionally, the preprocessing phase includes removing stop words, which are common words that do not add significant value to the analysis, such as “the", “and", “a", etc. However, we deliberately chose not to remove negative words like “against," “no," “not," “don’t," etc., since they play significant roles in sentiment meaning. Furthermore, the stemming technique was applied to reduce words to their root forms, while tokenization breaks sentences into individual words to improve the text analysis’s efficiency. These comprehensive preprocessing steps help transform the raw data into a clean and structured format and refine the text data for more accurate sentiment analysis. In addition, these formats are essential for effective analysis and decision-making.

### 3.3 TriLex

Our objective is to assess if the proposed TriLex model could help improve the prediction of unsupervised sentiment for short text. One popular way to perform sentiment analysis is through lexicon-based approaches, which rely on pre-built dictionaries that associate words with positive, negative, or neutral sentiment scores. TriLex is an unsupervised approach that leverages the majority votes of three sentiment analysis lexicons. It helps automate the clustering of opinions, emotions, and attitudes from short text without reliance on previously labeled sentiments.

The process involves clustering opinions expressed in the text based on the agreement among multiple lexicon-based sentiment analysis tools as a strong signal for sentiment classification while handling disagreement through a dynamic threshold mechanism.

Given a short text input, TriLex first obtains sentiment predictions from TextBlob, VADER, and AFINN lexicons. If the three lexicons agree on the sentiment label, TriLex assigns the majority vote as the final sentiment label, considering it a strong label. In cases where the three lexicons disagree, TriLex treats the input as a weak label and applies a dynamic threshold mechanism to generate a new sentiment label. The dynamic threshold is derived from the majority vote and a configurable threshold parameter, allowing for flexible adjustment based on the target domain or application.

#### 3.3.1 Preliminary sentiment label assignment

The preliminary label is assigned to the sentiment based on the three popular lexicons: TextBlob, VADER, and AFINN. Each lexicon predicts sentiment category individually for all given sentiments. We choose the thresholds of these lexicons according to the previous research suggestion. Previous research suggests that both TextBlob and AFINN employ a sentiment analysis system based on a threshold of the polarity score. Specifically, a sentiment with a polarity score  > 0 is labeled as positive, a sentiment with a polarity score  < 0 is labeled as negative, and a sentiment with a polarity score  = 0 is labeled as neutral. Conversely, VADER utilizes a slightly different method for assigning sentiment labels. VADER labels a sentiment as positive if (*CompoundScore* ≥ 0 . 05), neutral if (0 . 05 > *CompoundScore* > − 0 . 05), and negative if (*CompoundScore* ≤ − 0 . 05). Table 1 shows the threshold values of each lexicon.

**Table 1:** Selected thresholds of TextBlob, VADER, and AFINN lexicons.

	Positive	Neutral	Negative
**TextBlob**	*Polarity* > 0	*Polarity* = 0	*Polarity* < 0
**VADER**	*Compound* ≥ 0 . 05	0 . 05 > *Compound* > − 0 . 05	*Compound* ≤ − 0 . 05
**AFINN**	*Polarity* > 0	*Polarity* = 0	*Polarity* < 0

#### 3.3.2 Dynamic threshold for weak labels

Weak labels are determined based on the disagreement among the three lexicons. TriLex generates a new sentiment label based on a dynamic threshold derived from the majority vote and a configurable threshold. Despite the simplicity of lexicon-based approaches, their performance varies depending on the text’s domain and the dictionary’s quality. Therefore, this study proposes an enhanced lexicon-based sentiment analysis approach (TriLex) to improve the classification accuracy for unsupervised sentiment estimation. The three lexicons have different sentiment score scales. Therefore, we normalize sentiment scores to ensure that the scores are on a consistent scale, making them comparable and interpretable. Consequently, we normalize the sentiment score from each lexicon to bring them to the range  [ 0 , 1 ]  using Eq (1).


$$\begin{array}{ccc} NSS_{t_{n}^{L_{m}}}=\frac{SS_{n}+SS_{\text{Max}}}{SS_{\text{Max}}-SS_{\text{Min}}} & & \end{array}$$ (1)

Here, NSS means normalized sentiment score, $t_{n}^{L_{m}}$ means the *nth* sentiment of the lexicon ($L_{m}$), $SS_{n}$ means the sentiment score of *nth* sentiment in ($L_{m}$) lexicon, $SS_{\text{Max}}$ means the maximum sentiment score and $SS_{\text{Min}}$ is the minimum sentiment score in the lexicon ($L_{m}$).

The composite sentiment score is calculated by assigning the average weight for the normalized sentiment score using Eq (2),


$$\begin{array}{ccc} CSS_{i}^{k}=\overset{3}{\underset{m=1}{\sum }}NSS_{t_{n}^{L_{m}}}\times W_{L_{m}} & & \end{array}$$ (2)

where $CSS_{n}^{m}$ means the composite sentiment score of *i*–*th* sentiment and $W_{L_{m}}$ means the average weight of lexicon ($L_{m}$).

After computing the composite sentiment score, we set a new threshold to categorize sentiment into positive, neutral, and negative. Algorithm (1) illustrates the process of score normalization, composite score generation based on weighted calculations, and sentiment categorization for the TriLex approach. First, the maximum and minimum values for TextBlob polarity, VADER compound score, and AFINN score are obtained from the input dataframe that contains sentiment scores from the preliminary sentiment assignment. Then, sentiment scores are normalized utilizing Eq (1), which maps the scores to a range of  [ 0 , 1 ] , where 0 represents the minimum value, and 1 represents the maximum value.

**Algorithm 1. TriLex sentiment labeling algorithm.**



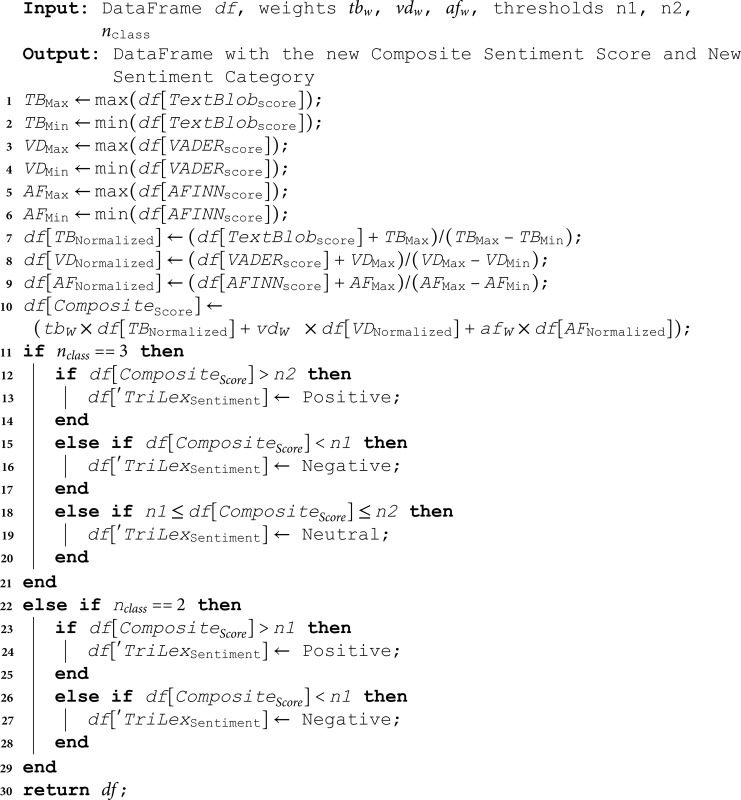



Next, the composite score is calculated by taking a weighted sum of the normalized score as shown in Eq (2), where $tb_{w}$, $vd_{w}$, and $af_{w}$ are the respective weights for TextBlob, VADER, and AFINN scores. To generalize our methodology, we make it applicable for two classes of classification (positive and negative) and three classes of classification (positive, negative, and neutral) based on the number of classes (n_class) and the specified threshold values (*n*1 and *n*2). For three classes of sentiments, the sentiment is assigned as “Negative" if Composite Score  < *n*1, “Neutral" if *n*1 ≤ *CompositeScore* < *n*2, and “Positive" if Composite Score  ≥ *n*2. On the other hand, for two classes of sentiment classification, the sentiment is assigned as “Negative" if Composite Score  < *n*1, and “Positive" otherwise.

For data points where all three lexicons (TextBlob, VADER, and AFINN) agree on the sentiment category, the composite score is calculated as the average of the TextBlob polarity and 1 (to map it to the range  [ 0 , 1 ] ). The sentiment category is assigned based on the agreed sentiment. For data points where the lexicons disagree on the sentiment categories, the composite score and sentiment category are calculated using the sentiment composite score described above in Eq (2). Finally, the new sentiment score and category are generated. This algorithm utilizes the majority vote among the TextBlob, VADER, and AFINN sentiment categories using a weighted approach, normalizes the scores to a common range, and classifies the sentiment into categories based on predefined thresholds, with the weights and thresholds being adjustable based on the specific requirements of the research.

Table 2 shows the values of the average weight of each lexicon and threshold values utilized in this study. The $TB_{W}$, $VD_{W}$, and $AF_{W}$ hyperparameters represent the average weights of TextBlob (20*%*), VADER (40*%*), and AFINN (40*%*), respectively. According to the experimental trail, the chosen threshold values are positive (*Score* > 0 . 5), neutral (0 . 4 ≤ *Score* ≤ 0 . 5), and negative (*Score* < 0 . 4) for the three classes. On the other hand, the threshold values for two classes were chosen as follows: positive (*Score* ≥ 0 . 75) and negative (*Score* < 0 . 75). The sensitivity to these hyperparameters is analyzed in the Results and Discussion section.

**Table 2:** Selected average weights and thresholds of the proposed TriLex approach.

# of Classes	$TB_{W}$	$VA_{W}$	$AF_{W}$	Positive	Neutral	Negative
**Three Classes (3)**	0 . 20	0 . 40	0 . 40	*Score* > 0 . 5	0 . 4 ≤ *Score* ≤ 0 . 5	*Score* < 0 . 4
**Two Classes (2)**	0 . 20	0 . 40	0 . 40	*Score* ≥ 0 . 75	—–	*Score* < 0 . 75

## 4 Results and discussions

To evaluate the effectiveness of the proposed TriLex methodology, we conducted experiments on the COVID-19 Vaccines dataset and three widely used benchmark datasets for sentiment analysis of the short text: Sentiment140, Amazon Review, and US Airline Tweets Sentiment. These datasets represent a diverse range of domains, including health-related discussions, social media, and product reviews, allowing us to assess the performance of TriLex across different contexts and sentiment expression patterns.

### 4.1 Experimental evaluation

We conducted experiments on four datasets described in Sect 3.1 from multiple domains to evaluate the performance of the proposed TriLex approach compared to relying on individual lexicons. We assess the performance using standard evaluation metrics for sentiment analysis tasks, including accuracy, precision, recall, and F1 score at two levels. First, we evaluate the performance of individual lexicons and TriLex with respect to the original label of the benchmark datasets. Moreover, we treat all datasets as unsupervised and assign the sentiment category based on TextBlob, VADER, AFINN, and the proposed TriLex. The performance is evaluated using the same standard evaluation metrics with respect to Logistic Regression, XGBoost, and LSTM.

Sentiment analysis has increasingly relied on word embedding techniques to capture textual features, with traditional methods like TF-IDF (Term Frequency-Inverse Document Frequency). TF-IDF can be useful in short text analysis by highlighting key terms that are uniquely present across different documents, which can be helpful in differentiating sentiment when short texts are distinct. Moreover, it remains relevant in settings where simplicity and interpretability are prioritized, as it relies solely on term statistics without requiring large corpora or extensive computational resources. However, TF-IDF does not capture semantic relationships or context offered by newer approaches such as Word2Vec and BERT. Word2Vec can enhance TF-IDF for short text by embedding words based on their surrounding words in a larger corpus, producing dense vectors that capture semantic similarity. However, because Word2Vec provides context-free embeddings (each word has a single vector representation), it may still struggle with word disambiguation in short texts where context is limited. BERT (Bidirectional Encoder Representations from Transformers) is particularly powerful for short text sentiment analysis, as it generates context-dependent embeddings by considering each word’s position and surrounding words. This ability allows BERT to manage the nuanced and often ambiguous language of short texts, providing better performance in capturing sentiment even with minimal data. BERT’s transformer-based architecture also mitigates the issue of sparsity by using self-attention mechanisms to highlight relevant text segments dynamically, making it highly effective for short text sentiment analysis compared to TF-IDF and Word2Vec. Therefore, we split the data into 80*%* of the data for training and 20*%* for testing. We address the class imbalanced issue by applying the under-sampling by removing completely at random examples that belong to the majority class. To evaluate predictive models, we use five-fold cross-validation to split the dataset.

To evaluate the performance of TriLex, an unsupervised sentiment analysis approach designed for short text, we employ three distinct machine learning models: Logistic Regression, XGBoost, and LSTM. Each of these models brings unique strengths to the task of sentiment classification, allowing for a comprehensive evaluation of TriLex’s effectiveness across different modeling approaches. Choosing these models for evaluating the proposed TriLex reflects a balanced strategy for capturing different aspects of sentiment through varied model strengths. Logistic Regression is a straightforward linear model suitable for binary or multiclass classification tasks, and it excels when the data has linearly separable classes. In the context of short text sentiment analysis, Logistic Regression can serve as a strong baseline for its interpretability and efficiency, providing a clear understanding of how specific words or features impact sentiment categorization. XGBoost is a powerful ensemble method that is capable to model complex and non-linear relationships without needing extensive feature engineering. Moreover, it can capture interactions among sparse and non-sequential features. LSTM is crucial for capturing temporal dependencies in sequences, making it well-suited for textual data where word order impacts sentiment. Although the unsupervised approach does not require labeled data, LSTM can effectively learn underlying sentiment patterns through sequence modeling in the short text by recognizing recurrent linguistic structures associated with the sentiment. LSTM networks are a type of recurrent neural network (RNN) specifically designed to capture dependencies in sequential data, which is particularly useful for text-based sentiment analysis. By processing words in sequence, LSTM models can account for contextual information in short texts, which enhances sentiment prediction accuracy. Applying LSTM to evaluate TriLex enables us to assess how well the lexicon-based features contribute to capturing the temporal dependencies in sentiment expressions, especially in texts where word order affects meaning.

These models offer a robust evaluation framework for TriLex. Logistic Regression provides a quick, interpretable baseline. XGBoost captures complex feature interactions and non-linearities and LSTM allows for the assessment of sequential patterns in short text. By comparing TriLex’s performance across these models, we can determine its adaptability and effectiveness in sentiment classification for diverse data characteristics and modeling requirements.

In a classification problem involving *n* different categories or classes, we can define various evaluation criteria to measure the performance of the model based on the true positive ($TP_{i}$), false positive ($FP_{i}$), true negative ($TN_{i}$), and false negative ($FN_{i}$) values obtained for each class *i*. The evaluation metrics used in conducted experiments are defined as follows.

1. Accuracy: measures the overall correctness of the predictions, calculated as the ratio of correctly classified instances to the total number of instances, as shown in Eq (3).
  $$\begin{array}{ccc} \text{Accuracy}=\overset{n}{\underset{i=1}{\sum }}\frac{TP_{i}+TN_{i}}{TP_{i}+TN_{i}+FP_{i}+FN_{i}} & & \end{array}$$ (3)
2. Precision: The ratio of true positive predictions to the total positive predictions, as shown in Eq (4).
  $$\begin{array}{ccc} \text{Precision (P)}=\overset{n}{\underset{i=1}{\sum }}\frac{TP_{i}}{TP_{i}+FP_{i}} & & \end{array}$$ (4)
3. Recall: The ratio of true positive predictions to the total actual positive instances, as shown in Eq (5).
  $$\begin{array}{ccc} \text{Recall (R)}=\overset{n}{\underset{i=1}{\sum }}\frac{TP_{i}}{TP_{i}+FN_{i}} & & \end{array}$$ (5)
4. Macro F1 Score: The macro-averaged of F1 Score, which is the harmonic mean of precision and recall, as shown in Eq (6).
  $$\begin{array}{ccc} \text{Macro F1 Score}=\overset{n}{\underset{i=1}{\sum }}\frac{2\times P_{i}\times R_{i}}{P_{i}\times R_{i}} & & \end{array}$$ (6)

### 4.2 Lexicons agreements and disagreements

**Fig 2:**
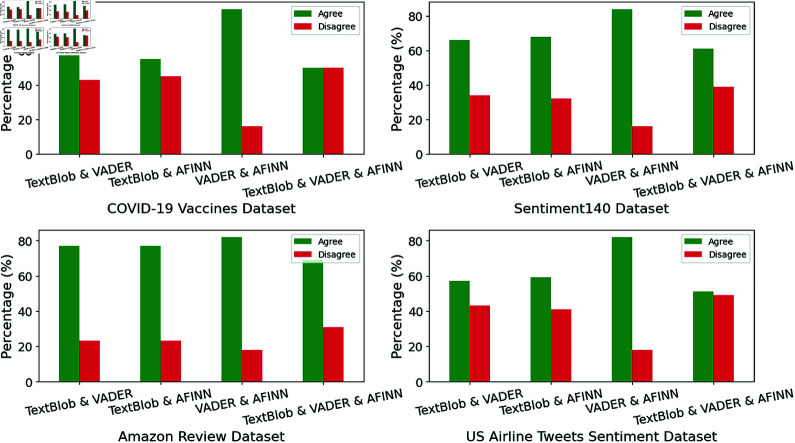
Agreement and disagreement distribution among TextBlob, VADER, and AFINN lexicons for sentiment categorization across four datasets.

The comparison of the three lexicons, TextBlob, VADER, and AFINN, demonstrates that VADER and AFINN agreed on approximately 82*%* − 84*%* of the sentiments category prediction for all datasets. However, TextBlob agreed with VADER and AFINN on roughly 55*%* − 77*%*. According to the agreements among the pair lexicons and experimental trail, we assigned weights to TextBlob, VADER, and AFINN as 20*%*, 40*%*, and 40*%*, respectively. Table 3 and Fig 2 illustrate the agreements and disagreements among the three lexicons in all datasets. The comparison of agreement and disagreement among TextBlob, VADER, and AFINN across various datasets highlights that VADER and AFINN consistently agree more closely than TextBlob. The higher agreement suggests that VADER and AFINN sentiment categorization methods may share underlying similarities or strengths in certain contexts, which TriLex leverages through its majority vote mechanism. The variations between columns (*TB* + *VD*, *TB* + *AF*, *VD* + *AF*, and *TB* + *VD* + *AF*) reflect how different lexicon combinations interact, which demonstrates TriLex’s adaptability in capturing diverse sentiment expressions.

**Table 3:** Agreement and disagreement among TextBlob (TB), VADER (VD), and AFINN (AF) for all datasets.

Dataset	TB+VD		TB+AF		VD+AF		TB+VD+AF	
**COVID-19 Vaccine**	Agree	57*%*	Agree	55*%*	Agree	**84%**	Agree	50*%*
	Disagree	43*%*	Disagree	45*%*	Disagree	16*%*	Disagree	50*%*
**Sentiment140**	Agree	66*%*	Agree	68*%*	Agree	**84%**	Agree	61*%*
	Disagree	34*%*	Disagree	32*%*	Disagree	16*%*	Disagree	39*%*
**Amazon Review**	Agree	77*%*	Agree	77*%*	Agree	**82%**	Agree	69*%*
	Disagree	23*%*	Disagree	23*%*	Disagree	18*%*	Disagree	31*%*
**US Airline Tweets**	Agree	57*%*	Agree	58*%*	Agree	**82%**	Agree	51*%*
	Disagree	43*%*	Disagree	42*%*	Disagree	18*%*	Disagree	49*%*

### 4.3 Performance of word embedding techniques on TriLex

We evaluated the efficiency of various word embedding techniques to assess their performance and computational complexity. Our findings indicate that TF-IDF is simple and interpretable, as it relies on simple term statistics without the need for large corpora or extensive computational resources. For example, TF-IDF extracted features in as little as 1 second for the Amazon Review and US Airline Tweets datasets and no more than 48 seconds for larger datasets like Sentiment140. In contrast, Word2Vec embeddings are slightly more computationally demanding, with feature extraction times ranging from 1 second for smaller datasets to 135 seconds for Sentiment140. However, BERT embeddings are significantly more resource-intensive, requiring approximately 4 days (259 , 320 seconds) for the COVID-19 Vaccines dataset and 5 days (432 , 420 seconds) for Sentiment140. Table 4 provides a detailed comparison of feature extraction times across these techniques.

**Table 4:** Time complicity of features extraction using different word embedding techniques.

Dataset	TF-IDF	Word2Vec	BERT
COVID-19 Vaccines	30 Seconds	115 Seconds	259 , 320 Seconds
Sentiment140	48 Seconds	135 Seconds	432 , 420 Seconds
Amazon Review	1 Second	1 Second	26 , 880 Seconds
US Airline Tweets	1 Second	1 Second	15 , 000 Seconds

The evaluation of multiple-word embedding techniques for TriLex across four datasets, including COVID-19 Vaccine, Sentiment140, Amazon Review, and US Airline Tweets, provides comprehensive insights into the trade-offs between accuracy, F1 score, and computational time for each method. The results in Table 5 show that BERT consistently outperforms TF-IDF and Word2Vec in terms of accuracy and F1 scores across all datasets and models. For instance, BERT achieves the highest F1 score of 98*%* with the LSTM model on both the COVID-19 Vaccine and Sentiment140 datasets. However, these gains come at a significant computational cost, with training times for BERT extending to 7 , 380 seconds on the COVID-19 Vaccine dataset and 11 , 280 seconds on Sentiment140 when paired with LSTM. In contrast, TF-IDF emerges as the most computationally efficient embedding technique, achieving feature extraction times as low as 1–36 seconds while maintaining competitive performance. Notably, TF-IDF paired with LSTM attains an F1 score of 98*%* on Sentiment140 and 88*%* on the US Airline Tweets dataset. Word2Vec offers reasonable performance in terms of accuracy and F1 score, which is lower than TF-IDF and BERT and reduces resource consumption.

The findings highlight that while BERT delivers state-of-the-art performance, its significant resource demands make it expensive for real-time or large-scale applications. On the other hand, TF-IDF is well-suited for scenarios where a computational efficiency is a priority, offering a balance between performance and resource consumption. These results underscore the importance of selecting embedding techniques based on the specific requirements of the task, including accuracy, scalability, and computational constraints.

**Table 5:** Training time and performance evaluation of multiple word embedding techniques for TriLex across various domains.

**COVID-19 Vaccine**									
	TF-IDF			Word2Vec			BERT		
**Model**	Accuracy	F1 Score	Time	Accuracy	F1 Score	Time	Accuracy	F1 Score	Time
LG	80*%*	80*%*	2 Sec	77*%*	77*%*	2 Sec	**87%**	**87%**	38 Sec
XGB	88*%*	88*%*	15 Sec	81*%*	80*%*	20 Sec	**93%**	**93%**	2 , 400 Sec
LSTM			3 , 180 Sec	91*%*	91*%*	5 , 880 Sec	**98%**	**98%**	7 , 380 Sec
**Sentiment140**									
	TF-IDF			Word2Vec			BERT		
**Model**	Accuracy	F1 Score	Time	Accuracy	F1 Score	Time	Accuracy	F1 Score	Time
LG	95*%*	95*%*	36 Sec	92*%*	92*%*	38 Sec	**97%**	**97%**	60 Sec
XGB	96*%*	96*%*	180 Sec	94*%*	93*%*	540 Sec	**96%**	**97%**	3 , 653 Sec
LSTM			6 , 780 Sec	96*%*	95*%*	9 , 480 Sec	**98%**	**98%**	11 , 280 Sec
**Amazon Review**									
	TF-IDF			Word2Vec			BERT		
**Model**	Accuracy	F1 Score	Time	Accuracy	F1 Score	Time	Accuracy	F1 Score	Time
LG	79*%*	77*%*	1 Sec	67*%*	67*%*	1 Sec	**85%**	**85%**	1 Sec
XGB	83*%*	82*%*	5 Sec	68*%*	68*%*	8 Sec	**87%**	**87%**	20 Sec
LSTM	85*%*	83*%*	28 Sec	78*%*	78*%*	120 Sec	**89%**	**88%**	420 Sec
**US Airline Tweets**									
	TF-IDF			Word2Vec			BERT		
**Model**	Accuracy	F1 Score	Time	Accuracy	F1 Score	Time	Accuracy	F1 Score	Time
LG	81*%*	81*%*	2 Sec	73*%*	73*%*	2 Sec	**89%**	**86%**	2 Sec
XGB	87*%*	87*%*	15 Sec	79*%*	79*%*	25 Sec	**91%**	**90%**	120 Sec
LSTM	88*%*	88*%*	36 Sec	82*%*	82*%*	180 Sec	**93%**	**93%**	540 Sec

### 4.4 Performance of TriLex and individual Lexicons in multiple datasets

**Fig 3:**
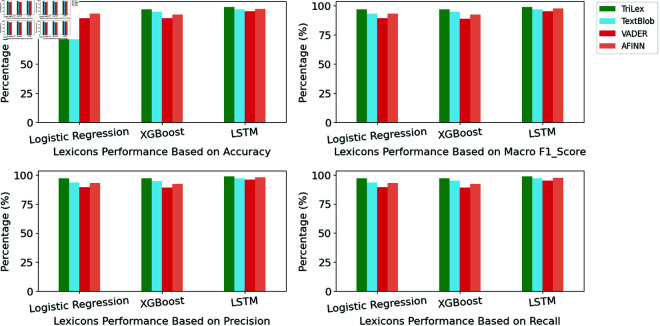
Performances of lexicons on the COVID-19 Vaccines dataset utilizing machine learning models and LSTM. The green bar represents the proposed TriLex approach, light blue represents TextBlob, red represents VADER, and DarkSalmon represents the AFINN lexicon.

The proposed TriLex approach is examined to determine the sentiment category for the COVID-19 Vaccines dataset [30,31]. To assess the effectiveness of the proposed TriLex approach, its performance is compared against individual lexicons using identical models and metrics discussed in Subsect 4.1. The comparative performance of the TriLex versus individual lexicons is visualized in Fig 3. Notably, across all models examined, the TriLex consistently exhibited higher accuracy, F1 score, precision, and recall when compared to individual lexicons. The proposed TriLex achieved an accuracy of 98 . 79*%*, F1 score 98 . 83*%*, precision 98 . 86*%*, and recall 98 . 79*%* with LSTM. The performances of lexicons with the Logistic Regression model illustrated that TriLex improves the evaluation metrics of the sentiment category by 4*%* − 8*%* compared to the individual lexicons.

The efficiency and robustness of our proposed TriLex approach are validated on three different datasets across multiple domains. These datasets are labeled; however, we utilize them as unsupervised. The performance is evaluated with similar metrics and models applied to the COVID-19 vaccine dataset. Furthermore, we compared the sentiment categories yielded by the individual lexicon and the TriLex with the original labels.

**Fig 4:**
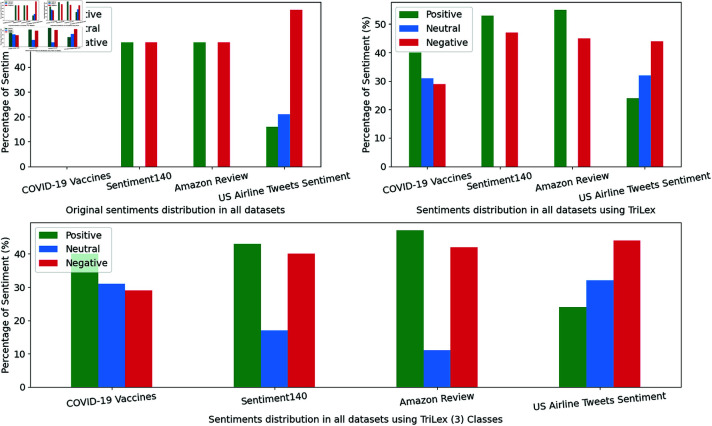
The original sentiment labels on the datasets (top left) vs. the generated labels using TriLex corresponding to the original label categories (top right). The bottom figure represents the sentiment distribution using TriLex considering three classes.

The Sentiment140 and Amazon Review datasets originally had two sentiment categories (positive and negative). Thus, when we considered these datasets as unsupervised, we assigned sentiment categories as original (positive and negative). Moreover, we examine the proposed TriLex approach to assign positive, negative, and neutral sentiment categories. Specifically, the Amazon Review dataset was labeled based on product rating, which may not reflect the true sentiment category. Therefore, we generate sentiment labels based on the review text. On the other hand, the US Airline Tweets Sentiment dataset has three label categories. We assigned three sentiment labels for the datasets that originally have two labels only to examine the efficiency and robustness of the proposed approach in capturing the sentiment class. Fig 4 demonstrates the distribution of the sentiment categories of the original sentiment label and the generated sentiment categories by applying the proposed TriLex approach.

The results of the evaluation of the sentiment categories follow the original datasets’ sentiment distributions generated leveraging the TriLex approach, which are shown in Table 6. Although XGBoost performs comparably to the proposed model using AFINN on the Sentiment140 dataset, our finding illustrates that the TriLex approach achieved the highest accuracy and F1 score in all datasets.

**Table 6:** The comparison of the average accuracy and average F1 score along with the standard deviation of TextBlob, VADER, AFINN, and the proposed TriLex approach. We test on three datasets: Sentiment140, Amazon Review, and US Airline Tweets Sentiment corresponding to the original labels.

Sentiment140								
	Baseline Approach						**Proposed Approach**	
**Approach**	TextBlob		VADER		AFINN		**TriLex**	
**Model**	Accuracy	F1 Score	Accuracy	F1 Score	Accuracy	F1 Score	Accuracy	F1 Score
LG	94*%* ∓ 0 . 02	94*%* ∓ 0 . 01	91*%* ∓ 0 . 01	90*%* ∓ 0 . 03	93*%* ∓ 0 . 03	93*%* ∓ 0 . 01	**97%** ∓ 0 . 01	**97%** ∓ 0 . 02
XGB	95*%* ∓ 0 . 02	95*%* ∓ 0 . 02	93*%* ∓ 0 . 03	93*%* ∓ 0 . 01	96*%* ∓ 0 . 01	96*%* ∓ 0 . 01	**96%** ∓ 0 . 02	**97%** ∓ 0 . 02
LSTM	96*%* ∓ 0 . 03	96*%* ∓ 0 . 03	97*%* ∓ 0 . 02	97*%* ∓ 0 . 01	96*%* ∓ 0 . 02	96*%* ∓ 0 . 02	**98%** ∓ 0 . 01	**98%** ∓ 0 . 01
**Amazon Review**								
	Baseline Approach						**Proposed Approach**	
**Approach**	TextBlob		VADER		AFINN		**TriLex**	
**Model**	Accuracy	F1 Score	Accuracy	F1 Score	Accuracy	F1 Score	Accuracy	F1 Score
LG	71*%* ∓ 0 . 02	70*%* ∓ 0 . 01	78*%* ∓ 0 . 03	77*%* ∓ 0 . 01	78*%* ∓ 0 . 02	77*%* ∓ 0 . 02	**85%** ∓ 0 . 01	**85%** ∓ 0 . 02
XGB	79*%* ∓ 0 . 02	77*%* ∓ 0 . 01	77*%* ∓ 0 . 03	77*%* ∓ 0 . 02	81*%* ∓ 0 . 02	80*%* ∓ 0 . 04	**87%** ∓ 0 . 03	**87%** ∓ 0 . 05
LSTM	80*%* ∓ 0 . 06	80*%* ∓ 0 . 03	82*%* ∓ 0 . 02	82*%* ∓ 0 . 02	82*%* ∓ 0 . 03	82*%* ∓ 0 . 01	**89%** ∓ 0 . 03	**88%** ∓ 0 . 02
**US Airline Tweets Sentiment**								
	Baseline Approach						**Proposed Approach**	
**Approach**	TextBlob		VADER		AFINN		**TriLex**	
**Model**	Accuracy	F1 Score	Accuracy	F1 Score	Accuracy	F1 Score	Accuracy	F1 Score
LG	79*%* ∓ 0 . 01	80*%* ∓ 0 . 01	76*%* ∓ 0 . 03	76*%* ∓ 0 . 01	80*%* ∓ 0 . 02	80*%* ∓ 0 . 01	**89%** ∓ 0 . 02	**86%** ∓ 0 . 02
XGB	81*%* ∓ 0 . 02	81*%* ∓ 0 . 01	75*%* ∓ 0 . 03	76*%* ∓ 0 . 03	83*%* ∓ 0 . 01	83*%* ∓ 0 . 01	**91%** ∓ 0 . 01	**90%** ∓ 0 . 02
LSTM	82*%* ∓ 0 . 02	83*%* ∓ 0 . 02	80*%* ∓ 0 . 02	80*%* ∓ 0 . 02	84*%* ∓ 0 . 04	85*%* ∓ 0 . 03	**93%** ∓ 0 . 04	**93%** ∓ 0 . 02

Additionally, we expand the sentiment labels for the Sentiment140 and Amazon Review datasets from their original binary categories (positive and negative) to include a third category, neutral. Table 7 presents the distribution of sentiment assignments across these three classes. Although the original datasets containing only positive and negative sentiments, applying the proposed TriLex approach demonstrated superior performance compared to individual lexicons. Specifically, TriLex improves the accuracy of sentiment prediction by 7*%* − 10*%* on the Amazon Review dataset.

To evaluate the significance of the improvement obtained by TriLex, we conducted statistical tests comparing the proposed TriLex with baseline lexicons using a paired t-test approach. We compute the *t* statistic and *p*-value based on accuracies for TriLex and each individual baseline lexicon. We choose the significance level *α* as *α* = 0 . 05 and identify an appropriate test statistic with a known probability distribution under the null hypothesis. The null hypothesis is rejected, and the improvement is deemed statistically significant at the 0 . 05 level if *p*-value is less than *α*. The statistical tests were performed on the results obtained from the LSTM model, as it achieved the highest accuracy across all datasets and sentiment analysis approaches.

**Table 7:** The comparison of the accuracy and F1 score of TextBlob, VADER, AFINN, and the proposed approach: TriLex. We test on Sentiment140 and Amazon Review datasets corresponding to the three labels (positive, neutral, and negative).

Sentiment140								
	Baseline Approach						**Proposed Approach**	
**Approach**	TextBlob		VADER		AFINN		**TriLex**	
**Model**	Accuracy	F1 Score	Accuracy	F1 Score	Accuracy	F1 Score	Accuracy	F1 Score
LG	65*%*	65*%*	59*%*	59*%*	64*%*	64*%*	**66%**	**66%**
XGB	65*%*	65*%*	59*%*	59*%*	62*%*	62*%*	**65%**	**65%**
LSTM	**69%**	**69%**	68*%*	68*%*	**69%**	**69%**	68*%*	69*%*
**Amazon Review**								
	Baseline Approach						**Proposed Approach**	
**Approach**	TextBlob		VADER		AFINN		**TriLex**	
**Model**	Accuracy	F1 Score	Accuracy	F1 Score	Accuracy	F1 Score	Accuracy	F1 Score
LG	67*%*	67*%*	49*%*	49*%*	58*%*	58*%*	**74%**	**74%**
XGB	64*%*	64*%*	57*%*	56*%*	57*%*	57*%*	**72%**	**71%**
LSTM	48*%*	44*%*	14*%*	18*%*	42*%*	50*%*	**60%**	**59%**

Our findings on the Sentiment140 dataset indicate that the *t* statistic for comparing TriLex with TextBlob and VADER is 2 . 8284, with a *p*-value of 0 . 0474. Additionally, the comparison of TriLex and AFINN yields a *t* statistic of 5 . 6569 and a *p*-value of 0 . 0048. For the Amazon Review dataset, the comparison between TriLex and TextBlob resulted in a *t* statistic of 3 . 5355 and a *p*-value of 0 . 0241, while the comparison between TriLex and VADER produced a *p*-value of 0 . 0000. The comparison between TriLex and AFINN resulted in a *t* statistic of 8 . 4853 and a *p*-value of 0 . 0011. In contrast, the results for the US Airline Tweets dataset showed that the comparisons between TriLex and both TextBlob and VADER yielded a *p*-value of 0 . 0000, while the comparison between TriLex and AFINN resulted in a *t* statistic of 1 . 4142 and a *p*-value of 0 . 2302. These statistical test results confirm that the improvement provided by TriLex is statistically significant at the 0 . 05 level.

The proposed TriLex significantly enhances the sentiment analysis lexicon-based approach, resulting in more accurate sentiment labels. Moreover, the consistent performance gains achieved by TriLex across diverse datasets and domains are attributed to the following key factors:

- By leveraging the collective features of multiple lexicons, TriLex can effectively capture a wider range of sentiment patterns and expressions compared to individual lexicons.
- The proposed dynamic threshold mechanism for handling weak labels (disagreement among lexicons) allows TriLex to adapt to the specific characteristics of the target domain or dataset, improving its ability to improve sentiment expressions.
- The majority vote and threshold approach employed by TriLex provides robustness against noise and outliers present in short text data, particularly in social media and informal contexts.

The empirical results and analysis demonstrate the effectiveness of the proposed TriLex methodology in improving sentiment analysis accuracy for short text across diverse domains and datasets. The ensemble-based approach, combined with a dynamic threshold, provides a scalable solution for unsupervised sentiment analysis and offers competitive performance compared to individual lexicon-based methods for unsupervised techniques.

### 4.5 Parameter sensitivity testing

The sensitivity of the results to hyperparameters is analyzed by adjusting individual parameters within predefined ranges while holding others constant, then observing and quantifying the resulting changes in model performance. We create a function that defines a process evaluating TriLex’s sentiment composite score to determine the optimal weights and classification thresholds. Moreover, it computes the accuracy and F1 score by comparing the labels generated by TriLex and LSTM to true labels. Hyperparameter ranges are specified for weights and thresholds, which are tested to find the optimal combination of *TB_W_*, *VD_W_*, *AF_W_*, *n*1, and *n*2 that maximizes model performance. This function evaluates various parameter combinations, identifies the best set based on accuracy, and outputs the optimal hyperparameters and their corresponding performance metrics. Our findings show that the best parameters for Sentiment140 datasets are *TB_W_* = 20%, *VD_W_* = 45%, *AF_W_* = 35%, and *n*1 = 0 . 82. On the other hand, the best parameters obtained for the Amazon Review dataset are *TB_W_* = 25%, *VD_W_* = 35%, *AF_W_* = 40%, and *n*1 = 0 . 80. For the US Airline Tweets dataset, which contains three labels, positive, neutral, and negative, the best parameters are *TB_W_* = 20%, *VD_W_* = 40%, *AF_W_* = 40%, *n*1 = 0.55, and *n*2 = 0 . 35. Therefore, we conclude that the threshold used in Table 2 in Sect 3 could be appropriate.

**Fig 5:**
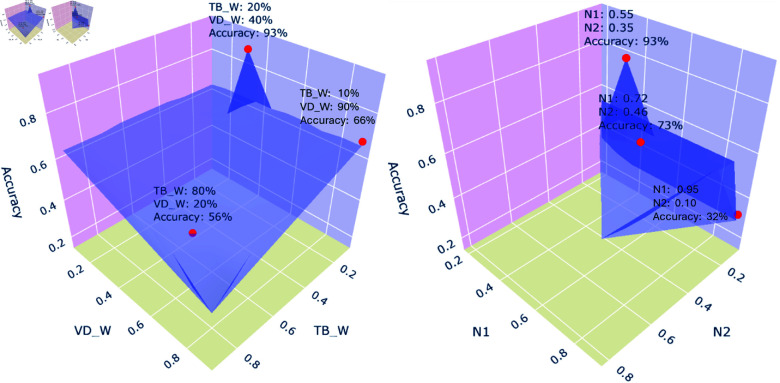
Parameter sensitivity analysis for the proposed TriLex used for the US Airline Tweets dataset. The left panel shows the accuracy as a function of $TB_{W}$ and $VD_{W}$ parameters while the right panel is visualizing the influence of *n*1 and *n*2 parameters on the accuracy.

Fig 5 presents a three-dimensional visualization of the parameter sensitivity analysis conducted for TriLex on the US Airline Tweets dataset. The left panel contains $TB_{W}$ and $VD_{W}$ parameters illustrating their optimal weight combinations concerning accuracy. The right panel showcases the best combination between *n*1 and *n*2 parameters. It is important to note that the parameters in the right panel are contingent upon the optimal values identified in the left panel, specifically when $TB_{W}=20\%$, $VD_{W}=40\%$, and $AF_{W}=40\%$. The $AF_{W}$ parameter is not plotted here because it is represented by the subtraction of TextBlob and VADER weights.

## 5 Conclusion

In this paper, we present TriLex, a novel approach for unsupervised sentiment analysis of short text. It treats the majority vote agreement among lexicons as a strong label and disagreement through a dynamic threshold mechanism. TriLex aims to improve the accuracy and robustness of sentiment predictions for short text. The unsupervised nature of TriLex makes it scalable and adaptable to new domains where labeled data is limited or unavailable. The experimental results on COVID-19 Vaccines and benchmark datasets demonstrate the competitive performance of the TriLex approach, highlighting its potential for practical applications in domains where labeled data is scarce, or sentiment expressions are dynamic.

However, it is important to acknowledge that TriLex’s performance, like any unsupervised method, can be influenced by the quality and coverage of the underlying lexicons used. Although we utilize widely used and well-established lexicons in our experiments, exploring the incorporation of domain-specific or dynamically generated lexicons could potentially further enhance the accuracy of our approach. Future work could explore the incorporation of additional lexicons or sentiment analysis techniques into the ensemble and the development of adaptive threshold mechanisms appropriate to specific domains or languages.
